# *Physcomitrella patens DCL3* Is Required for 22–24 nt siRNA Accumulation, Suppression of Retrotransposon-Derived Transcripts, and Normal Development

**DOI:** 10.1371/journal.pgen.1000314

**Published:** 2008-12-19

**Authors:** Sung Hyun Cho, Charles Addo-Quaye, Ceyda Coruh, M. Asif Arif, Zhaorong Ma, Wolfgang Frank, Michael J. Axtell

**Affiliations:** 1 Department of Biology, Pennsylvania State University, University Park, Pennsylvania, United States of America; 2 Department of Computer Science and Engineering, Pennsylvania State University, University Park, Pennsylvania, United States of America; 3 Plant Biology Graduate Program, Pennsylvania State University, University Park, Pennsylvania, United States of America; 4 Plant Biotechnology, Faculty of Biology, University of Freiburg, Freiburg, Germany; 5 Integrative Biosciences Graduate Program in Bioinformatics and Genomics, Pennsylvania State University, University Park, Pennsylvania, United States of America; Washington University School of Medicine, United States of America

## Abstract

Endogenous 24 nt short interfering RNAs (siRNAs), derived mostly from intergenic and repetitive genomic regions, constitute a major class of endogenous small RNAs in flowering plants. Accumulation of *Arabidopsis thaliana* 24 nt siRNAs requires the Dicer family member DCL3, and clear homologs of *DCL3* exist in both flowering and non-flowering plants. However, the absence of a conspicuous 24 nt peak in the total RNA populations of several non-flowering plants has raised the question of whether this class of siRNAs might, in contrast to the ancient 21 nt microRNAs (miRNAs) and 21–22 nt *trans*-acting siRNAs (tasiRNAs), be an angiosperm-specific innovation. Analysis of non-miRNA, non-tasiRNA hotspots of small RNA production within the genome of the moss *Physcomitrella patens* revealed multiple loci that consistently produced a mixture of 21–24 nt siRNAs with a peak at 23 nt. These *Pp23SR* loci were significantly enriched in transposon content, depleted in overlap with annotated genes, and typified by dense concentrations of the 5-methyl cytosine (5 mC) DNA modification. Deep sequencing of small RNAs from two independent *Ppdcl3* mutants showed that the *P. patens DCL3* homolog is required for the accumulation of 22–24 nt siRNAs, but not 21 nt siRNAs, at *Pp23SR* loci. The 21 nt component of *Pp23SR*-derived siRNAs was also unaffected by a mutation in the RNA-dependent RNA polymerase mutant *Pprdr6*. Transcriptome-wide, *Ppdcl3* mutants failed to accumulate 22–24 nt small RNAs from repetitive regions while transcripts from two abundant families of long terminal repeat (LTR) retrotransposon-associated reverse transcriptases were up-regulated. *Ppdcl3* mutants also displayed an acceleration of leafy gametophore production, suggesting that repetitive siRNAs may play a role in the development of *P. patens*. We conclude that intergenic/repeat-derived siRNAs are indeed a broadly conserved, distinct class of small regulatory RNAs within land plants.

## Author Summary

Very small RNAs (between ∼21 and ∼30 single-stranded bases) are a ubiquitous component of gene regulation in nearly all eukaryotic organisms. The small RNA repertoire of angiosperms (the flowering plants) is exceptionally diverse and includes conspicuous populations of 21 nt microRNAs, as well a diverse set of 24 nt short, interfering RNAs (siRNAs). The 24 nt siRNAs have well-documented roles in enforcing the silence of parasitic regions of the genome, but are not readily apparent in the small RNA populations of several lineages of ancient, non-flowering plants. We found numerous “hotspots” of small RNA production from the genome of the moss *P. patens* that produced a mix of 21–24 nt siRNAs. Except for their broad mix of sizes, these hotspots were reminiscent of the 24 nt siRNA loci of angiosperms: they tended to associate with decayed transposons, to avoid annotated genes, and to be densely modified with the epigenetic mark 5-methyl cytosine. Deletion of a *P. patens* Dicer gene abolished production of 22–24 nt siRNAs both from these loci and transcriptome-wide, especially from repetitive regions. We conclude that both microRNAs and intergenic/repeat-associated siRNAs are ancient small RNA regulators in plants, but that the sizes of the siRNAs themselves have drifted over time.

## Introduction

Most eukaryotes analyzed to date express diverse small silencing RNAs which direct the sequence-specific repression of target RNAs. Small silencing RNAs are bound to Argonaute or Piwi proteins, which modulate target expression by a variety of molecular mechanisms [1]; specificity of targeting is mediated by RNA-RNA base-pairing between small RNA and target, while repression is mediated either directly or indirectly by the associated Argonaute or Piwi protein.

Two major types of small silencing RNAs have been described in plants: MicroRNAs (miRNAs), and short interfering RNAs (siRNAs). miRNAs are ubiquitous regulators of gene expression in animals, plants, and some unicellular eukaryotes. Most plant miRNAs are 21 nts in length and are defined by precise excision from a single-stranded, stem-loop precursor by the action of a Dicer protein. Mature miRNAs often function to repress the expression of an evolved set of protein-coding mRNA targets. miRNAs regulate thousands of mRNAs in animals and have had a profound impact upon the evolution of 3′-untranslated regions [2]–[4], which harbor many miRNA target sites. Plant miRNA targets seem to be less numerous, but many of them are critical for development and other processes [5]. Endogenous siRNAs have also been extensively characterized in *Arabidopsis thaliana*, where they are processed by Dicer proteins from long, perfectly double-stranded RNA (dsRNA) precursors. The endogenous dsRNA precursors are most often produced by RNA-dependent RNA polymerases (RDRs). The majority of expressed small RNAs in *A. thaliana* depend on the activity of two RDR proteins [6]–[8], implying that siRNA production from RDR-dependent dsRNA precursors is rampant in plants.

Plant Dicers (known as DCLs for “Dicer-Like”), Argonautes (AGOs) and RDRs are all encoded by multi-gene families; in *A. thaliana* specific family members are specialized for distinct endogenous small RNA producing pathways. DCL1 and AGO1 are required for the accumulation and function of most miRNAs [9]–[11], which in plants are almost uniformly 21 nts in length. miRNA accumulation has not been reported to require an RDR, consistent with origins from single-stranded primary transcripts. RDR6 and DCL4 produce a minority of endogenous siRNAs [6]; these are typically secondary siRNAs referred to as *trans*-acting (tasiRNAs) and are produced after small RNA-mediated cleavage of a primary transcript. tasiRNAs are mostly 21 nts in length, but small amounts of 22 nt tasiRNAs are typically observed as well [6],[12]. Finally, RDR2, DCL3, and AGO4 conspire to produce and utilize 24 nt siRNAs [13],[14]. The induction of *A. thaliana* 24 nt siRNAs correlates with the *de novo* deposition of repressive DNA and histone modifications [14],[15]; genome-wide, 24 nt siRNAs are enriched in intergenic regions and within repetitive elements, where they have been suggested to function to maintain transcriptional repression [7],[16].

The small RNA population of wild-type *A. thaliana* shows two distinct peaks at 21 nts and 24 nts in length [17], with the latter composed almost exclusively of *DCL3*-dependent siRNAs [7]. However, small RNA populations from non-angiosperm species, including mosses [18]–[20], lycopods [21], and gymnosperms [22],[23] conspicuously lack an obvious population of 24 nt species, which raised the question of whether the *DCL3*-dependent 24 nt siRNA pathway might have been a derived feature of angiosperm species. However, when the abundant 21 nt miRNAs and 21–22 nt tasiRNAs are subtracted, the remaining small RNAs from the moss *Physcomitrella patens* show a broader size distribution of 21–24 nt species [21]. Coupled with the existence of a clear *DCL3* homolog, these observations prompted us to search for intergenic/repetitive siRNA-producing loci in *P. patens*. Here, we identified *P. patens* loci which produced a mix of 21–24 nt siRNAs from primarily intergenic and repetitive regions of the genome, and which were densely populated by the 5-methyl cytosine (5 mC) DNA modification. Using deep sequencing of small RNAs from *Ppdcl3* deletion mutants, we observed that *PpDCL3* was required for the accumulation of 22–24 nt siRNAs, but not 21 nt siRNAs, from these loci. Loss of *PpDCL3*-dependent siRNA accumulation correlated with the de-repression of two abundant long terminal repeat (LTR) retrotransposon-associated reverse transcriptase families. Unlike in *A. thaliana*, *P. patens dcl3* mutants also demonstrated developmental abnormalities suggesting that repetitive siRNAs contribute to moss development. These observations demonstrate that a specialized, *DCL3*-dependent siRNA production system associated with transposons and other non-genic regions of the genome is an ancestral feature of land plants, although the sizes of the relevant siRNAs can differ between lineages.

## Results

### Two Classes of Small RNA Producing Loci in *P. patens*

Readily identifiable miRNAs and tasiRNAs account for a minority of expressed *P. patens* small RNAs [21]. We therefore sought to annotate other types of small RNA expressing regions of the *P. patens* genome by identifying loci corresponding to small RNA production “hotspots”. A previously reported dataset of expressed *P. patens* small RNAs [12] was first filtered to remove any small RNAs corresponding to previously annotated *P. patens MIRNA* hairpins or tasiRNA loci. We then ranked genomic loci for their small RNA producing activity based on the number of reads observed for exactly matched small RNAs. To account for the uncertainty of the genomic origins of small RNAs whose sequences matched multiple genomic loci, reads were repeat-normalized by dividing by the number of exact matches between the small RNA and the genome [24],[25].

**Figure 1:**
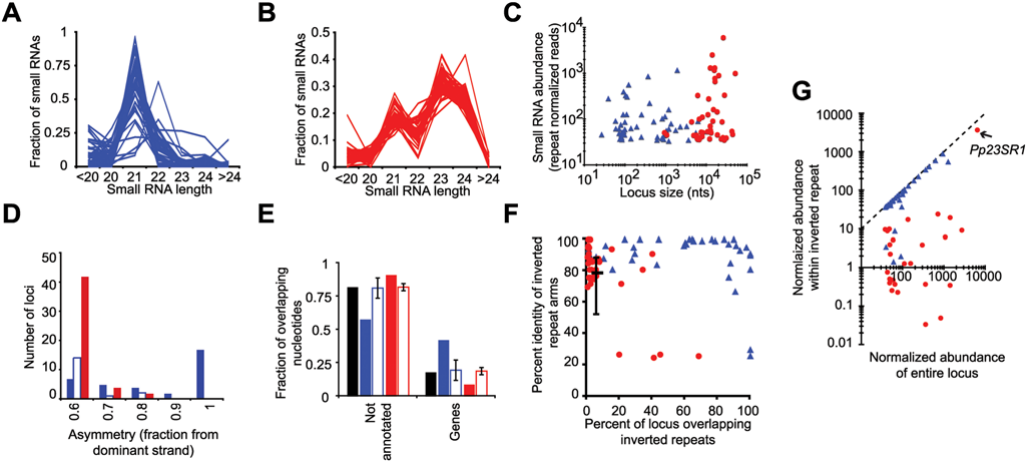
Two classes of small RNA producing loci in the moss *P. patens*. (A) Distribution of small RNA lengths within the 52 of the 100 most prolific, uncharacterized small RNA producing loci dominated by 21 nt small RNAs. Each line represents the small RNA size distribution within a single locus. These are referred to as the *Pp21SR* loci. (B) As in A for the 48 of the 100 most prolific small RNA producing loci which were typified by a collection of 21–24 nt small RNAs. These are referred to as the *Pp23SR* loci. (C) Scatterplot depicting the normalized small RNA abundances vs. the sizes of the small RNA producing loci. Blue triangles, *Pp21SR* loci; red circles, *Pp23SR* loci. (D) The fraction of the normalized small RNA abundance emanating from the most abundant genomic strand is categorized for *Pp23SR* loci (red) and *Pp21SR* loci (blue). The subset of the *Pp21SR* loci where more than 50% of the abundance stemmed from small RNA dyads (where the same small RNA mapped to both genomic strands within a locus) are indicated by hollow blue bars, while solid blue bars indicate *Pp21SR* loci with less than 50% dyad abundances. None of the *Pp23SR* loci had a majority of small RNA abundance derived from such dyads. (E) Fractions of the genome (black), *Pp21SR* loci (blue), *Pp23SR* loci (red) and randomized controls (hollow bars) overlapping annotated *P. patens* genes. (F) Percent identity of inverted repeat arms vs. the percent of *Pp21SR* (blue) and *Pp23SR* (red) loci which those arms overlap. Black indicates percentage of the *P. patens* genome which overlaps inverted repeats, with error bars indicating the first quartile, median, and third quartiles of genome-wide inverted repeat identities. (G) Normalized small RNA abundance within inverted repeat stems vs. the total normalized abundance for *Pp21SR* (blue) and *Pp23SR* (red) loci. For loci which overlapped multiple inverted repeats, the one with the maximum abundance is shown. The position corresponding to *Pp23SR1* is highlighted.

The top 100 non-miRNA, non-tasiRNA small RNA producing regions of the *P. patens* genome clearly fell into two distinct classes, initially discerned based on the lengths of the associated small RNAs: Those which were dominated by RNAs 21 nts in length, and those producing a mixture of 21–24 nt RNAs in a strikingly consistent ratio (Figures 1A–B). These two types of small RNA producing loci were dubbed the *Pp21SR* (21 nucleotide Small RNA) and *Pp23SR* (21, 23, and 24 nucleotide Small RNA) loci, respectively. Most loci (89 out of 100) had at least one corresponding small RNA which uniquely mapped to the genome (Tables S1, S2, and S3), confirming that they were sources of small RNA accumulation. These 100 loci were almost evenly split between the *Pp21SR* class and the *Pp23SR* class with 52 loci in the former and 48 loci in the latter. The two classes were also differentiated by size: The *Pp23SR* loci generally spanned larger genomic regions between ∼5,000 nts and ∼50,000 nts in length (median = 11,902 nts), while the *Pp21SR* loci were mostly between 100 and 1,000 nts in length (median = 247.5 nts; Figure 1C). A fundamental distinction between different Dicer-derived small RNAs is the nature of their precursors. Successive processing of long, perfectly base-paired dsRNAs is the defining feature of siRNAs. In contrast, precise processing of the stem regions of single-stranded stem-loop structures defines miRNA biogenesis, while more diverse cohorts of small RNAs can arise from chaotic processing of other single-stranded stem-loop precursors such as *A. thaliana IR71* [16]. Provided that a large enough number of small RNAs have been sequenced from a particular locus, distinguishing dsRNA-derived siRNAs from stem-loop-derived small RNAs is straightforward: The former will have approximately equal numbers of small RNAs matching both strands of the genome, while small RNAs from the latter will be confined to one strand or the other. We found that almost all of the *Pp23SR* loci had an approximately equal small RNA abundance corresponding to both genome polarities, suggesting that this class largely consisted of siRNAs derived from dsRNA precursors (Figure 1D). In contrast, many of the *Pp21SR* loci had pronounced strand asymmetry suggestive of processing from a single-stranded precursor. The two classes were also distinguished based upon their overlaps with annotated gene products: Relative to the genome as a whole and to randomized control cohorts, *Pp21SR* loci were enriched for annotated gene content while *Pp23SR* loci were depleted (Figure 1E).

Loci which initially appeared to produce equal amounts of small RNAs from both genomic polarities might in fact be regions transcribed to form perfect or near-perfect single stranded stem-loop RNAs. If this were the case, many or all of the corresponding small RNA sequences would have matched exactly twice within the locus (once to each strand of the genome), thus leading to the erroneous conclusion that they were derived from a long dsRNA precursor instead of a single-stranded, stem-loop precursor. Many of the *Pp21SR* loci, but none of the *Pp23SR* loci, had more than half of their normalized abundances accounted for by such ambiguously mapped small RNA dyads (Figure 1D). Thus, the most prolific *Pp21SR* loci in *P. patens* chiefly produced single-stranded small RNA precursors, while the *Pp23SR* loci were clearly templates for the production of long dsRNA precursors which are processed into siRNAs.

Genome-wide analysis revealed that 6.2% of the *P. patens* genome was contained within inverted repeats (Figure 1F). The arms of these 132,656 inverted repeats had a median identity of 79%. Most of the small RNA clusters (71% of the *Pp21SR* loci and 85% of the *Pp23SR* loci) had at least some overlap with one or more inverted repeats. However, the percentage of nucleotides which overlapped with inverted repeats for most of the *Pp23SR* loci was close to the genome-wide value of 6.2% (Figure 1F). This indicated that there was no specific enrichment. In contrast, most of the *Pp21SR* loci were clearly enriched for inverted repeat content relative to the genome as a whole; in many cases the majority of the nucleotides within *Pp21SR* loci were contained within the arms of inverted repeats (Figure 1F). If an inverted repeat were truly causal in the production of small RNAs from a given locus, most or all of the observed small RNA abundance would be expected to map to the two arms of a single repeat unit; this would reflect processing of the helical portion of the stem-loop RNAs which result from transcription of inverted repeats. For many of the *Pp21SR* loci, but none of the *Pp23SR* loci, all of the observed small RNAs mapped to the arms of a single inverted repeat (Figure 1G). Detailed examination of the *Pp21SR* loci revealed that they were comprised of several distinct types, including previously un-annotated miRNAs (Table S1), heterogenously processed inverted repeats of variable length, a few apparent siRNA clusters, and some loci which defied classification (Table S2).

### *Pp23SR* Loci Primarily Derive from LTR-Retrotransposons and *Helitron* DNA Transposons

**Figure 2:**
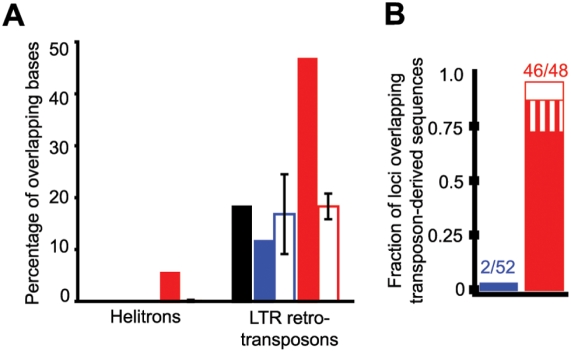
*Pp23SR* loci are enriched in transposon-derived sequences. (A) The percentage of nucleotides with high similarity to *Helitron* rolling circle DNA transposons (left) and LTR-retrotransposons (right) is shown for the *P. patens* genome as a whole (black), the 52 *Pp21SR* loci (blue-filled), and the 48 *Pp23SR* loci (red-filled). The averages and standard deviations for ten cohorts of *Pp21SR* loci (blue-hollow) and *Pp23SR* (red-hollow) with randomized positions in the genome are also shown. (B) The fraction of *Pp21SR* (blue) and *Pp23SR* (red) loci which had at least some overlap with sequences similar to LTR-retrotransposons (filled), *Helitrons* (hollow), or both (vertical stripes) is shown.

The genome of *P. patens* is dominated by interspersed repetitive elements derived from multiple rounds of LTR-retrotransposon invasions [26]. Using relatively strict protein-based similarity searches against known plant transposons (CENSOR using TBLASTX; [27]) we found that 19.4% of the draft genome sequence had significant similarity to known interspersed repetitive elements. Almost all of the similarities were to LTR-retrotransposons (18.8%) with minor contributions from *Helitron* rolling circle DNA transposons (0.12%), and other elements (Figure 2A). Collectively, 12.0% of the nucleotides within the 52 *Pp21SR* loci overlapped regions similar to LTR-retrotransposon proteins, while there was no overlap with other types of interspersed elements (Figure 2A). This level of overlap was also observed with cohorts of randomized control loci. On a locus by locus basis, only two of the 52 *Pp21SR* loci overlapped with regions similar to LTR-retrotransposon proteins, and none overlapped regions similar to *Helitrons* (Figure 2B). These data indicate that the *Pp21SR* loci are not enriched for interspersed repetitive elements relative to the genome as a whole. In contrast, 47.3% and 6.0% of the nucleotides within the 48 *Pp23SR* loci overlapped with regions similar to LTR-retrotransposon and *Helitron* elements, respectively. These were both significant enrichments (p≪0.001, one-sided Z-test) as judged by values obtained using randomized cohort loci (Figure 2A). Only two of the 48 *Pp23SR* loci did not have any overlap with either of these two elements; the remaining 46 either overlapped regions similar to LTR-retrotransposons, *Helitrons*, or both (Figure 2B). The association of many of the *Pp23SR* loci with LTR-retrotransposons was independently supported by using LTR_FINDER to find intact elements based upon long terminal repeat identification and the presence of target site duplications [28]; several of the *Pp23SR* loci, but none of the *Pp21SR* loci, were in regions predicted to correspond to intact LTR-retrotransposon elements (Tables S2, S3). Thus, we conclude that *Pp23SR* loci, but not *Pp21SR* loci, almost exclusively arise from transposon-derived interspersed repetitive elements.

**Figure 3:**
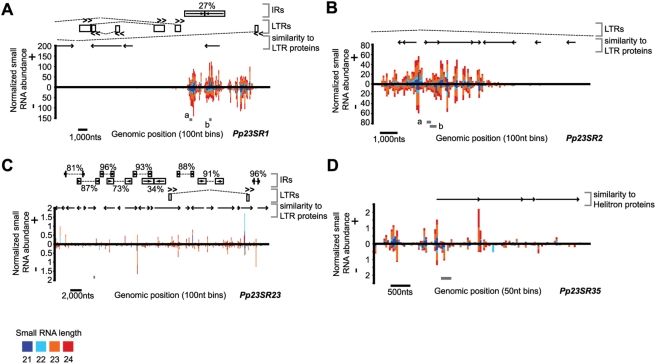
Examples of *Pp23SR* loci. Details can be found in Table S3. (A) Schematic of small RNA accumulation at *Pp23SR1*. The normalized abundances of corresponding small RNAs between 21 and 24 nts in length were plotted relative to the genomic sequence as a function of the positions of their 5′ ends using a bin size of 100 nts. “+” indicates small RNAs matching the positive strand of the genome, and “−” indicates small RNAs matching the minus strand. Relative positions of sequence features are indicated. Percentages refer to identities between inverted repeat arms. Dashed lines connect predicted LTRs but do not indicate full-length, intact elements. Gray boxes show positions of PCR amplicons used for methylation analyses. (B) Schematic of *Pp23SR2*, displayed as in A. (C) Schematic of *Pp23SR23*, displayed as in A. (D) Schematic of *Pp23SR35*, displayed as in A, except using 50 nt bins.

The most active of the *Pp23SR* loci, *Pp23SR1*, was centered upon an area of nested LTR-retrotransposons (Figure 3A). Almost all of the small RNAs from this locus originated from a region between two different LTRs situated in a convergent orientation. The long terminal repeats of intact LTR-retrotransposons contain strong PolII promoters which drive expression of transposon genes – thus, the arrangement of the *Pp23SR1* locus suggested that a trigger dsRNA could have been produced by convergent transcription. The presence of a long, low identity inverted repeat in this region was also noted (Figure 3A). Secondary structure predictions indicate that this inverted repeat does not possess sufficient self-homology to form a stem-loop structure in either polarity; thus it is unlikely that the siRNAs from this region were processed directly from a single-stranded stem-loop precursor. Most of the other *Pp23SR* loci did not share these characteristics: *Pp23SR2* produced small RNAs from a region with several areas of similarity to LTR-retrotransposon proteins, and was contained within the widely separated LTRs of a predicted intact element (Figure 3B). Nearly the entirety of the *Pp23SR23* locus had similarity to LTR-retrotransposon proteins, and was covered by many inverted repeats of varying identities (Figure 3C). However, the observed patterns of small RNA accumulation from *Pp23SR23* did not show any obvious relationship to these features. The *Pp23SR35* locus appears to be a *Helitron* DNA transposon (Figure 3D), and also had no obvious trigger for the initiation of small RNA production.

### *PpDCL3* Affects the Rate of Gametophore Production

We hypothesized that the *Pp23SR*-associated siRNAs were analogous to the *DCL3*-dependent 24 nt intergenic/repetitive siRNAs of *A. thaliana*. To test this hypothesis, we deleted the *P. patens DCL3* homolog using homologous recombination (Figure S1A). Four individual transformed plants were isolated in which PCR analysis confirmed the precise integration of the deletion cassette into the *PpDCL3* locus by homologous recombination (Figure S1B). In all four lines, *PpDCL3* expression was reduced to levels not detectable by RT-PCR (Figure S1C). DNA blot analysis demonstrated that two of the four (*Ppdcl3*-5 and *Ppdcl3*-10) had a single integration of the knock-out construct only at the targeted locus (data not shown); these two lines were used for all further studies.

**Figure 4:**
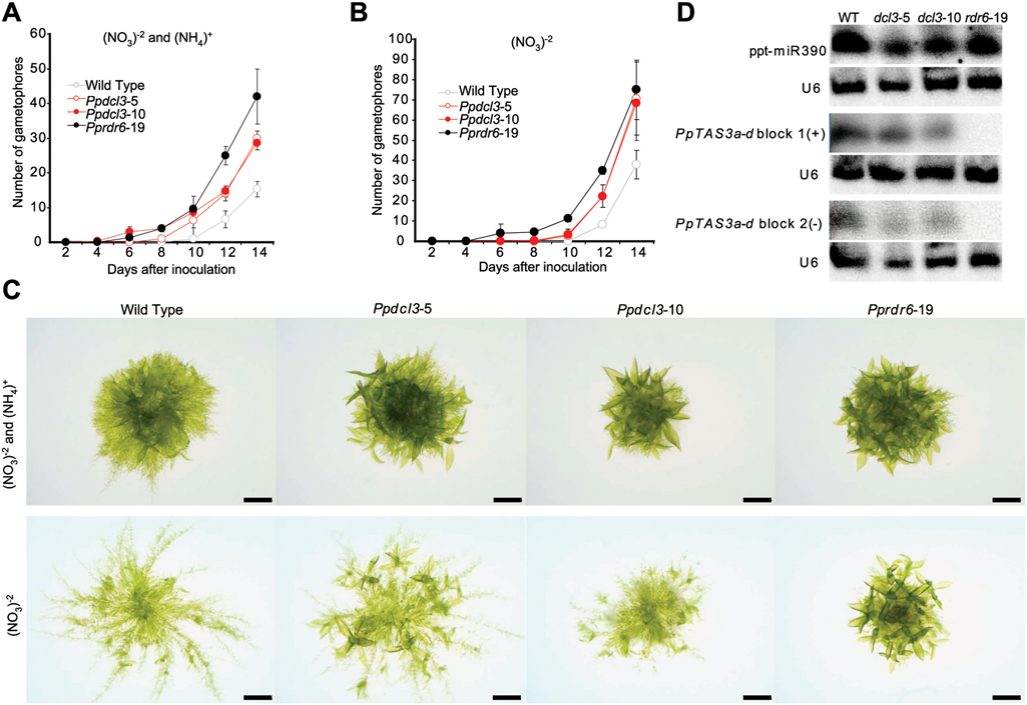
Deletion of *PpDCL3* accelerates gametophore production. Two-day old protonemata (1–2 mm in diameter) from the indicated strains were inoculated onto media with (A) or without (B) ammonium supplementation and scored for gametophore number every other day. Error bars indicate standard deviation (n = 9). (C) Representative colony morphologies after 12 days of growth. Bars indicate 1 mm. (D) RNA gel blot analysis for the indicated small RNAs. U6 snRNA was used as a loading control.

Preliminary observations indicated that *Ppdcl3* mutants produced gametophores faster than the wild type, which reminded us of the previously reported *Pprdr6* phenotype [29]. Thus, we directly compared the timing of gametophore production in *Ppdcl3*, *Pprdr6*, and the wild type in detail. Similar sizes of protonemata were inoculated onto minimal media with or without ammonium supplementation and monitored for gametophore number over a fortnight. Regardless of ammonium availability, the rate of gametophore production in *Ppdcl3* mutants was accelerated relative to wild-type (Figures 4A–B). However, this rate was also clearly less than that observed in the *Pprdr6* mutant. The absence of ammonium accelerates gametophore development and encourages growth of caulonemal filaments in *P. patens* [30], as illustrated by comparing wild-type colony morphologies after 12 days (Figure 4C). Under these conditions, the difference between the *Ppdcl3* and *Pprdr6* phenotypes was dramatically highlighted: *Ppdcl3* plants largely retained the extensive production of caulonema which was lost in *Pprdr6* plants (Figure 4C). At the molecular level, *Pprdr6* mutants fail to accumulate miR390-dependent tasiRNAs from *PpTAS3a–d* [29]. In contrast, RNA blots indicated that *Ppdcl3* mutation had at best a very minor effect on accumulation of *PpTAS3a–d* tasiRNAs (Figure 4D). Together, these observations demonstrated that the developmental and molecular phenotypes of *Ppdcl3* and *Pprdr6* mutants were similar, but distinct. Furthermore, in contrast to *A. thaliana dcl3* mutants *Ppdcl3* plants have a readily apparent developmental defect.

### *Ppdcl3* Mutants Are Impaired in siRNA Accumulation from *Pp23SR* Loci

The impact of *Ppdcl3* mutation upon small RNA accumulation was tested by deep sequencing of small RNAs. Two *Ppdcl3*-derived small RNA libraries were constructed; one each from *Ppdcl3*-5 and *Ppdcl3*-10 plants. As controls, we also generated and sequenced a wild-type library and a library from the *Pprdr6*-19 mutant [29]. All RNA samples were harvested from 10-day old protonemata grown and harvested concurrently under identical conditions. Wild-type, *Ppdcl3*, and *Pprdr6* libraries each used a different 3′ linker sequence to allow the libraries to be mixed prior to sequencing. Two separate channels of an Illumina Genome Analyzer were used to sequence two mixtures of the four libraries: Run 1 contained wild-type, *Ppdcl3*-5, and *Pprdr6*-19 while Run 2 contained wild-type, *Ppdcl3*-10, and *Pprdr6*-19 (Table 1). This design created two technical replicates for wild-type and *Pprdr6*-19 small RNAs as well as two *Ppdcl3* samples from independent alleles. After parsing adapter sequences, reads which matched the sense strand of *P. patens* rRNAs and/or which failed to exactly match the *P. patens* version 1.1 draft genome assembly were discarded, resulting in over 700,000 genome-matched reads per genotype (Table 1).

**Table 1:** New *P. patens* small RNA libraries (NCBI GEO GSE12468).

Run	Library	Unique Sequences	Reads
1	Wild Type (replicate 1)	122,572	367,957
1	*Ppdcl3*-5	66,175	525,027
1	*Pprdr6*-19 (replicate 1)	107,996	380,210
2	Wild Type (replicate 2)	128,740	388,750
2	*Ppdcl3*-10	55,771	394,560
2	*Pprdr6*-19 (replicate 2)	103,908	362,774

**Figure 5:**
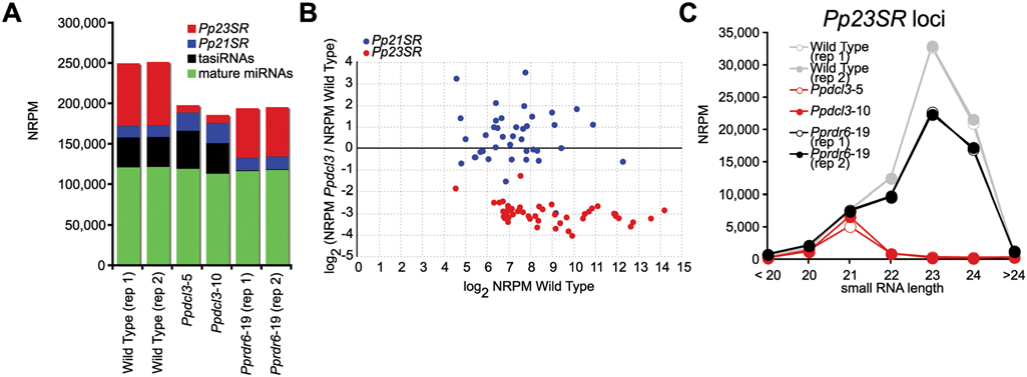
*PpDCL3* is required for the accumulation of 22–24 nt siRNAs from *Pp23SR* loci. (A) Abundance of sequenced small RNAs matching annotated small RNA loci from the indicated samples. NRPM: Normalized reads per million. (B) The ratio of the mean NRPM from the *Ppdcl3* samples over the mean of the two wild type replicates is plotted for the indicated loci as a function of mean abundance in the wild type. (C) Small RNA abundance from *Pp23SR* loci binned by small RNA length.

Data normalization allowed assessment of the impact of *Ppdcl3* and *Pprdr6* mutations on different classes of small RNA loci. Overall mature miRNA accumulation was not noticeably affected in either *Ppdcl3* or *Pprdr6* mutants (Figure 5A). As previously reported [29], tasiRNA expression was essentially eliminated in the *Pprdr6* small RNA sample; in contrast, *Ppdcl3* mutants had no discernable effect upon tasiRNA expression levels. *Ppdcl3*, but not *Pprdr6* samples had a strong reduction in *Pp23SR*-derived siRNA expression, but not a complete elimination (Figure 5A). This reduction in overall *Pp23SR* siRNA accumulation was not the result of just a few loci: All individual *Pp23SR* loci accumulated fewer small RNAs in the *Ppdcl3* samples relative to wild-type (Figure 5B). The *Ppdcl3*-dependent reduction in *Pp23SR*-derived siRNA accumulation was due to the almost complete loss of 22–24 nt siRNA accumulation (Figure 5C). However, the levels of *Pp23SR*-derived 21 nt siRNAs were unaffected by deletion of *PpDCL3* – these residual 21 nt siRNAs were also not dependent on *PpRDR6* function. Both the *PpDCL3*-dependent 22–24 nt siRNAs and the *PpDCL3*-independent 21 nt siRNAs from *Pp23SR* loci tended to have 5′ A or U residues (Figure S2). It is possible that, as in *A. thaliana* [31]–[33] this tendency reflected the binding preferences of one or more *P. patens* AGO proteins. Alternatively, this AU 5′ nucleotide bias could have been due to siRNA strand selection based on thermodynamic asymmetry as initial siRNA strands with 5′ A∶U pairs would tend to be more weakly paired in initial siRNA duplexes [34],[35].

### *PpDCL3* Is Required for the Accumulation of 22–24 nt siRNAs from Repetitive Genomic Regions

**Figure 6:**
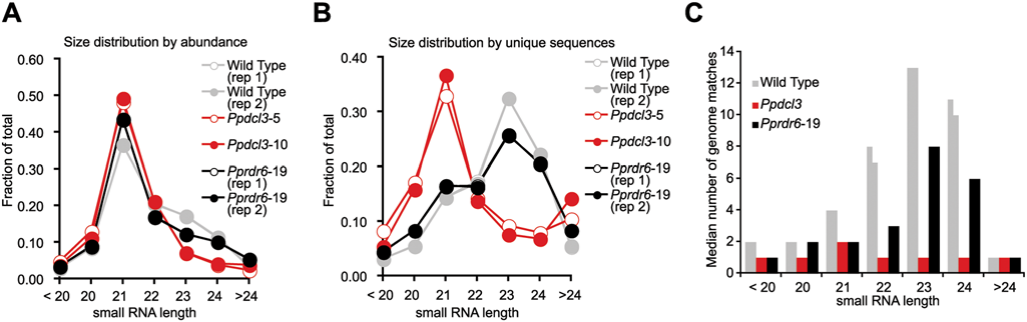
Transcriptome-wide effects of *PpDCL3* deletion on 23–24 nt RNA accumulation. (A) Size distribution of sequenced small RNAs, counted by abundance (number of reads). (B) Size distribution of sequenced small RNAs, counted by uniquely obtained sequences, regardless of their apparent abundance. (C) Histogram displaying median number of genome matches for sequenced small RNAs from the indicated samples. Replicates are shown as two identically colored bars.

As observed in prior samples of *P. patens* small RNAs, a plurality of small RNA abundance was accounted for by 21 nt species; this was also true in both the *Ppdcl3* and *Pprdr6* samples (Figure 6A). However, the shoulder of 23 and 24 nt small RNAs present in the wild-type was clearly diminished in the *Ppdcl3* small RNA samples (Figure 6A). This effect was dramatically highlighted when the small RNA populations were counted based upon distinct sequences regardless of the number of reads, thus strongly diminishing the contribution of highly abundant sequences (mostly miRNAs) to the overall profile. Viewed in this way, both the wild-type and *Pprdr6* samples had diverse 23 and 24 nt RNA populations which were lost in the *Ppdcl3* samples (Figure 6B). Most of the *Ppdcl3* defect in 23–24 nt RNA accumulation was attributable to the loss of small RNAs with a 5′ A or U residue (Figure S3). Thus, *PpDCL3* is required for the accumulation of a substantial amount of all 23–24 nt RNAs expressed by *P. patens*. *Pprdr6* samples also showed a slight decrease in 23–24 nt RNA accumulation relative to the wild-type, suggesting *PpRDR6* might make a minor contribution to *PpDCL3* function (Figures 6A–B). In the wild-type samples, 22, 23, and 24 nt RNAs tended to match multiple sites in the genome (Figure 6C), indicating that small RNAs of this length are more likely to match repetitive sequences. This trend was not evident in the *Ppdcl3* samples, where the median number of genome matches for the remaining 22–24 nt RNAs was reduced to one (Figure 6C). Thus, *PpDCL3* is required for the transcriptome-wide accumulation of 22–24 nt RNAs which tend to match repetitive regions. The possibility that *PpRDR6* might make a minor contribution to the accumulation of these repetitive 22–24 nt RNAs was also suggested by a reduction in the median number of genome hits observed in the *Pprdr6* samples (Figure 6C).

### Maintenance of Dense Cytosine Methylation at *Pp23SR* Loci Is Largely Unaffected by *Ppdcl3* Mutation

**Figure 7:**
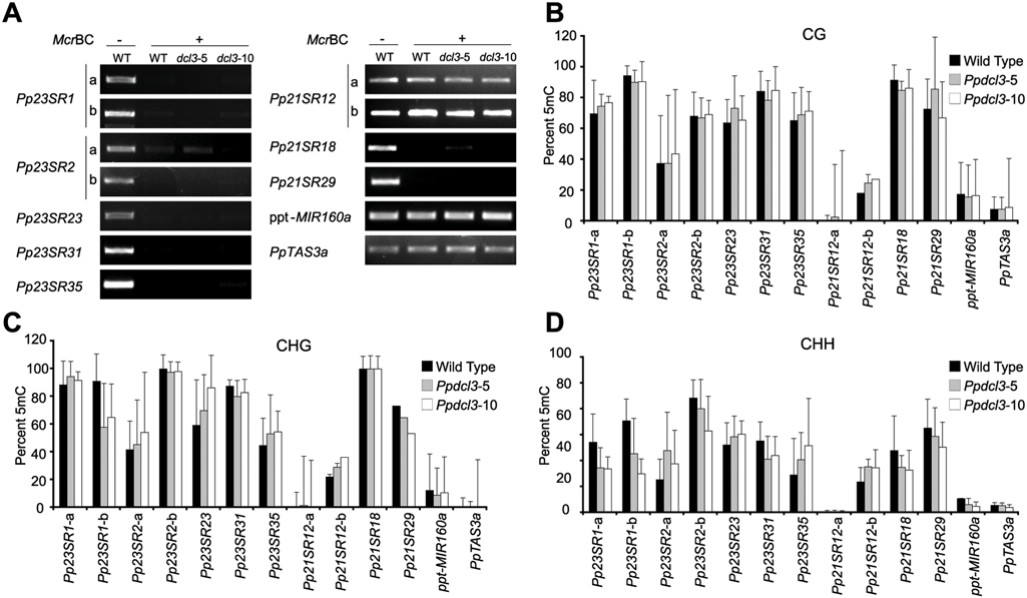
Dense 5-methyl cytosine modification at *Pp23SR* loci is largely unaffected by deletion of *PpDCL3*. (A) Amplification of the indicated loci from genomic DNA pre-treated with (+) or without (−) the 5-methyl cytosine specific endonuclease *Mcr*BC. WT: Wild Type. (B) The fraction of methylated cytosines in the symmetric CG context within the indicated loci was estimated by sequencing PCR products derived from bisulfite-converted genomic DNA. 5 mC: 5-methyl cytosine. Error bars indicate standard deviation. (C) As in B for cytosines in the symmetric CHG context. (D) As in B for cytosines in the asymmetric CHH context. Details of bisulfite sequencing data are given in Table S5. For all panels, where present, letters indicate distinct PCR products from different regions of a locus.

*A. thaliana DCL3*-dependent 24 nt small RNAs correlate with chromatin modifications at the encoding loci [13], [14], [36]–[38]. These 24 nt small RNAs are important in directing asymmetric cytosine methylation at endogenous, transcriptionally silenced loci [39]. Because the *Pp23SR* loci were dominated by 23 and 24 nt siRNAs dependent on a *DCL3* homolog, we tested whether they also correlated with 5 mC deposition. We first employed an assay based on the methylation-sensitive endonuclease *Mcr*BC. *Mcr*BC can digest 5 mC-modified DNA in both symmetric and asymmetric contexts; thus, diminished or no amplification relative to an undigested control sample is indicative of 5 mC modification [40]. All of the tested *Pp23SR* loci were sensitive to *Mcr*BC treatment, indicating that *Pp23SR* loci were highly methylated (Figure 7A). In contrast to *Pp23SR* loci, *Mcr*BC digestion had little to no effect on the amplification of *Pp21SR12* regions nor on ppt-*MIR160a* or *PpTAS3a*. Only two of the 52 *Pp21SR* loci overlapped with transposons (Figure 2B); both of these outliers (*Pp21SR18* and *Pp21SR29*) were also densely methylated indicating that 5 mC deposition at small RNA loci is not strictly limited to *Pp23SR* loci (Figure 7A). As assayed by *Mcr*BC analysis, none of these methylation patterns were substantially affected in either *Ppdcl3* mutant.

Sequencing of PCR products derived from bisulfite treated genomic DNA was performed to examine 5 mC patterns at these loci at single-base resolution (Figures 7B–D). Consistent with the *Mcr*BC data, all of the *Pp23SR* loci analyzed had high concentrations of 5 mC. Importantly, considerable 5 mC was observed in all contexts, including the non-symmetric CHH (Figure 7D); 5 mC in this context cannot be retained after DNA replication via maintenance methyltransferases acting on hemimethylated daughter strands and must instead be maintained by a different cue. 5 mC in the CHH context was not unique to *Pp23SR* loci, as demonstrated by the dense CHH modifications at the transposon-associated *Pp21SR18* and *Pp21SR29* loci (Figure 7D). In contrast, the *Pp21SR12* locus had much lower levels of 5 mC in all contexts (Figures 7B–D). Because a single sample of DNA per genotype was used for the amplifications, the low proportion of 5 mC for *Pp21SR12* served as an internal control for bisulfite conversion efficiency. Similarly, ppt-*MIR160a* and *PpTAS3a* also had very low levels of 5 mC regardless of the context. 5 mC densities in the CG context were not substantially affected by *Ppdcl3* mutation (Figure 7B). An approximately 30% reduction in CHG methylation was observed in *Pp23SR1*-b region in both *Ppdcl3* mutants, though CHG methylation in the *Pp23SR1*-a region was unchanged. In addition, CHH methylation in both *Pp23SR1* regions was reduced in *Ppdcl3* mutants, as was CHH methylation of *Pp23SR2*-b and *Pp23SR31*. However, non-CG methylation at other loci was either unchanged or slightly increased in the two *Ppdcl3* mutants. Thus, we conclude that while *PpDCL3*-dependent siRNAs may be responsible for the maintenance of a small portion of non-CG 5 mC modifications at individual loci, they are generally not necessary to maintain overall 5 mC patterns regardless of context.

### *PpDCL3* Suppresses Expression of LTR-Retrotransposon Associated Reverse Transcriptases

**Figure 8:**
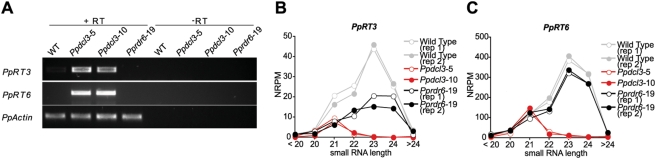
*PpDCL3* suppresses LTR retrotrasnsposon-associated reverse transcriptases. (A) RT-PCR for the indicated reverse transcriptase families was performed using cDNA from the indicated strains. Templates prepared without addition of reverse transcriptase (-RT) were used as a control for DNA contamination. *PpActin* was used as a positive control. (B) Total small RNA abundance from the 452 genomic sites corresponding to the potential *PpRT3* amplicons, binned by length. NRPM: Normalized reads per million. (C) As in B for the 426 potential *PpRT6* amplicons.

We hypothesized that *PpDCL3*-dependent siRNAs could serve to repress expression of homologous transcripts. Thus, we attempted to detect RNA accumulation from *Pp23SR* loci by RT-PCR. Primers specific for several top *Pp23SR* loci were generally unable to amplify any transcripts, regardless of genotype (Figure S4). We next used LTR_STRUC [41] to find and classify putative reverse transcriptase (RT) domains from relatively intact *P. patens* LTR retrotransposons. Oligos for several RT families (which we dubbed *PpRT1* to *PpRT6*) were designed and used for RT-PCR. Transcripts for *PpRT3* and *PpRT6* were not detectable in wild type but significantly accumulated in *Ppdcl3*-5 and *Ppdcl3*-10 (Figure 8A). The *PpRT3* and *PpRT6* transcripts had many hundreds of possible origins in the genome; tabulation of all matching small RNAs from these dispersed loci demonstrated that, in the wild type, they displayed a similar profile as the *Pp23SR* hotspots (Figures 8B–C). Importantly, *PpRT3* and *PpRT6* associated 22–24 nt small RNAs were absent in *Ppdcl3* mutants. These data indicate that *PpDCL3* is required to repress expression of at least a subset of LTR retrotransposons, likely via 22–24 nt siRNA production.

## Discussion

### Diversity of *P. patens* Small RNA Loci

We found several classes of small RNA producing loci in *P. patens*. Among the heterogenous *Pp21SR* loci, inverted-repeat derived 21mers were found in a continuum which ranged from the precisely processed miRNAs to more chaotically processed inverted repeats of much more variable size. An identical spectrum of inverted-repeat derived small RNAs has been described in *A. thaliana* [16],[25]. At one end of the spectrum, the miRNAs have well understood molecular and biological roles in the regulation of target genes *in trans*. However, the biological functions of the less precisely processed inverted-repeats remain unknown. It's possible that some of these inverted-repeats represent an intermediate state of miRNA evolution [42], while others may have currently unknown functions, or perhaps have no function at all. The biogenesis of some of the *P. patens Pp21SR* loci remains obscured. Some have an accumulation pattern which resembles a miRNA/miRNA* duplex but without apparent inverted repeats or predicted stem-loop secondary structures, while others may be siRNAs derived from a dsRNA precursor. *P. patens* also expresses mixtures of 21–24 nt siRNAs from broad genomic regions, which we call *Pp23SR* loci to reflect the peak of 23 nt RNA abundance. *Pp23SR* loci were depleted in gene content and enriched in overlap with apparent transposable elements.

### Homologous *DCL3*s Are Required for Different Sized siRNAs

Biochemical and genetic evidence has demonstrated that the individual *A. thaliana* Dicers each produce small RNAs of one or two sizes: DCL1 produces 21 nt miRNAs, DCL2 produces 22–23 nt siRNAs, DCL3 produces 24 nt siRNAs, and DCL4 produces 21 nt siRNAs [13],[43],[44]. *Pp23SR* loci were reminiscent of *A. thaliana DCL3*-dependent 24 nt siRNA hotspots in their tendency towards intergenic regions, accumulation of small RNAs predominately greater than 21 nts in size, and frequent co-occurrence with transposable elements. This analogy was strengthened by the demonstration that most *Pp23SR* siRNAs were lost upon deletion of the *PpDCL3* locus. Thus, we conclude that *Pp23SR* loci are analogous in function to the strictly 24 nt-producing loci of *A. thaliana*.

Unexpectedly, *PpDCL3* was required for the accumulation of a broad size range of siRNAs. At the *Pp23SR* loci, *Ppdcl3* mutants were nearly devoid of 22–24 nt siRNAs; transcriptome-wide, repetitive 22–24 nt RNAs were strongly diminished in this mutant. This contrasts with *AtDCL3*, which produces strictly 24 nt siRNAs *in vitro* [44] and which is defective only in 24 nt RNA accumulation *in vivo* [7]. It is possible that *PpDCL3* also strictly produces one size of siRNA. Under this scenario, the defects in accumulation of other sizes of small RNAs could be due to the activity of other Dicers dependent upon a facilitating role played by *PpDCL3*. Alternatively, it may that *PpDCL3* directly produces RNAs of three different sizes. Further experiments will be needed to differentiate between these two hypotheses. Regardless of the size differences between *A. thaliana* and *P. patens DCL3*-dependent siRNAs, our data clearly indicate that intergenic and repetitive siRNA production is an ancestral land plant trait. Thus, the possible loss or replacement of *DCL3* function in gymnosperms [22] must be a derived state.

*P. patens* appears to lack a *DCL2* homolog [21]. In one sense, *PpDCL3* could be said to combine the roles of *AtDCL2* (which produces 22 and 23 nt siRNAs) and *AtDCL3* (which produces 24 nt siRNAs) by directing the accumulation of 22–24 nt siRNAs. However, *AtDCL2*-dependent siRNA loci do not coincide with *AtDCL3*-dependent siRNA loci and, unlike *PpDCL3*, *AtDCL2* makes only a very small overall contribution to the overall siRNA population [7]. The fact that *AtDCL3* is clearly not the most closely related plant Dicer to *AtDCL2* [21],[45] also argues against the hypothesis that *DCL2* arose from an ancestral *DCL3* gene.

### Functional Redundancy at *Pp23SR* Loci

Deletion of *PpDCL3* ablated 22–24 nt siRNA production from *Pp23SR* loci but had no effect upon 21 nt siRNAs. Thus, we hypothesize that a second DCL also acts in conjunction with *PpDCL3* to produce *Pp23SR*-derived siRNAs. Besides *PpDCL3*, the *P. patens* genome contains one *DCL4* homolog and two *DCL1* homologs [21]. Because *AtDCL4* is required for chiefly 21 nt tasiRNA, secondary siRNA, and viral siRNA accumulation, we suspect that its moss homolog produces the residual 21 nt RNAs at the *Pp23SR* loci. Interestingly, these residual 21 nt siRNAs are not dependent upon *PpRDR6*, whose *A. thaliana* homolog is closely associated with *DCL4* function. Instead, *PpRDR6* partially contributed to 22–24 nt small RNA accumulation in *Pp23SR* loci. This functional redundancy is not exactly the same as that previously reported at *A. thaliana* 24 nt siRNA loci: In *A. thaliana*, the products of other DCLs only become apparent upon loss of *DCL3* function [7],[43], while in *P. patens* they are present even in the wild-type.

All *Pp23SR* loci assayed had dense 5 mC modifications in all contexts. This further solidifies the connection of the *Pp23SR* loci to the 24 nt siRNA loci of *A. thaliana*. It is possible that, as in *A. thaliana* [15], the siRNAs generated at *Pp23SR* loci can direct *de novo* cytosine methylation in the asymmetric CHH context. However, complete removal of *PpDCL3* function and the consequent loss of all 22–24 nt siRNAs at *Pp23SR* loci did not substantially affect the maintenance of wild-type 5 mC patterns in most instances examined. It may be that the residual 21 nt siRNAs which persist at *Pp23SR* loci in the *Ppdcl3* background are sufficient to maintain 5 mC deposition, buffering the effect of the *Ppdcl3* mutation. The fact that the two transposon-associated *Pp21SR* loci, which are naturally devoid of *PpDCL3*-dependent 22–24 nt siRNAs, also possessed dense 5 mC modifications in all contexts is consistent with this idea. Alternatively, it is possible that *PpDCL3*-dependent siRNAs are critical for the establishment, but not the maintenance, of 5 mC patterns at the *Pp23SR* loci and perhaps elsewhere. Indeed, components of the 24 nt siRNA pathway are required at the *A. thaliana SDC* locus to initiate 5 mC deposition in all contexts, but not to maintain these patterns once established [46]. Similarly, ablation of 24 nt siRNAs via *dcl3* mutation does not substantially affect the maintenance of 5 mC deposition at several other *A. thaliana* loci [47]. Some well-studied *A. thaliana* loci where 5 mC density is affected in *dcl3* mutants (*AtSN1*, *FWA*; [15],[47]) have very weak production of 24 nt siRNAs in the wild-type (in contrast to the siRNA hotspots we examined in *P. patens*), while maintenance of 5 mC density is only weakly diminished at the *MEA-ISR* and 5S rDNA loci in *A. thaliana dcl3* mutants [13],[15],[48]. Thus, the lack of strong effects upon 5 mC maintenance at *Pp23SR* loci in the *Ppdcl3* mutants is consistent with previous observations from *A. thaliana*. A third possibility is that the 5 mC and siRNAs associated with *Pp23SR* loci reflect independent processes which act in parallel to maintain the silence of the affected genomic regions.

### Biological Roles of *PpDCL3*

*Atdcl3* mutants lose most 24 nt repetitive siRNA accumulation [7] but have not been reported to display obvious developmental abnormalities [13]. In contrast we found that *Ppdcl3* plants, which lacked 22–24 nt repetitive siRNAs, displayed an accelerated production of gametophores relative to the wild type. While this developmental acceleration is less severe than that of *Pprdr6* plants, several lines of evidence suggest that *PpDCL3* and *PpRDR6* might have partially overlapping roles: Both mutants have similar developmental abnormalities, *TAS3* tasiRNA accumulation is slightly decreased in *Ppdcl3* mutants, and *Pprdr6* mutants slightly impact the production of repetitive 22–24 nt siRNAs. The *Ppdcl3* developmental phenotype is unlikely to be the result of stochastic epialleles because it is identical in multiple, independently-derived mutants and does not require several generations to become manifest. One possibility is that slight tasiRNA defects in *Ppdcl3* are responsible for the developmental phenotype. Alternatively, a *PpDCL3*-dependent small RNA, such as a miRNA with unusual biogenesis requirements, could directly regulate genes critical for gametophore development. It is also possible that the repetitive siRNAs themselves, or the suppression they seem to confer on certain repetitive elements, plays a role in moss development.

Production of 22–24 nt siRNAs from the most abundant *Pp23SR* “hotspots” was drastically reduced in *Ppdcl3* mutants. However, neither the maintenance of 5 mC DNA modifications nor the transcriptional suppression of these hotspots were substantially altered in *Ppdcl3* mutants. However, transcript accumulation of two highly abundant LTR retrotransposon-associated RT families was strongly enhanced by deletion of *PpDCL3*, despite the persistence of *PpDCL3*-independent 21 nt siRNAs. Thus, *PpDCL3* is necessary for the suppression of at least some highly repetitive elements, most likely as a direct result of 22–24 nt siRNAs. It is important to emphasize that the transcriptional reactivation of RT domains observed in *Ppdcl3* mutants does not necessarily correlate with re-activation of transposition-competent elements – we have not directly tested for transposition. However, the data do suggest that the repetitive 22–24 nt siRNAs may indeed play important roles in repressing transposition and defending genome integrity. We conclude that *AtDCL3*-derived, repetitive siRNAs are, like miRNAs and tasiRNAs, a distinct class of specialized small RNAs with ancient origins within the land plants.

## Materials and Methods

### Data Sources and Accessions

*P. patens* expressed small RNAs from [12] (NCBI GEO accession GSE5103) were filtered to eliminate sequences corresponding to miRNA hairpins annotated in miRBase (version 10.0; [49]) as well as those corresponding to the *PpTAS3a–d* loci (accessions BK005825–BK005828). Draft assembly version 1.1 of the *P. patens* genome was used throughout [26], as was the “filtered models 3” set of gene annotations. *P. patens* miRNAs used in the analysis of the new *P. patens* small RNAs were from miRBase version 11.0. Newly generated small RNA datasets were deposited with NCBI GEO (GSE12468).

### Discovery of Small RNA-Expressing Loci

Small RNAs from GSE5103 were mapped to the *P. patens* draft genome assembly version 1.1 using NCBI blastall version 2.2.17 (non-default parameters: -p blastn -e 0.1 -F F -W 6 -m 8 -v 100000 -a 2) and filtered to retain only exact matches. The repeat-normalized magnitude of each small RNA's expression from a given locus was calculated by dividing the number of times a given small RNA was sequenced by the total number of exact matches in the genome [25]. Genomic regions expressing high levels of small RNAs were found by analyzing these repeat-normalized scores in 500 nt bins across the genome as described by [25]. The boundaries of the regions represented the positions of the terminal small RNAs in the cluster.

### Annotation of Interspersed Repetitive Elements and Inverted Repeats

Standalone CENSOR (version 4.2.8; [27]) was used to find protein-based similarities to curated plant repetitive elements with the non-default parameters -ns –tr –nofound –nomasked –lib pln. The dataset of plant repetitive elements, plnrep.ref, was derived from the September 24, 2007 RepBase update [50]. The command-line version of LTR_FINDER version 1.02 [28] was used to find full length long terminal repeat retrotransposons with the default parameters except that the maximum LTR size was set to 100,000 nts and a target-site duplication was required (-D 100000 –F 00001000000). Inverted repeats were found using einverted (from the 5.0.0 version of EMBOSS; [51]) using default parameters except that the maximum repeat length was set to 5000.

### Targeted Disruption of *PpDCL3*

The disruption construct was designed to replace the entire ORF of *PpDCL3* with a CaMV35S promoter-*hptII*-CaMV terminator, hygromycin-resistance cassette. Two sets of primers (PpDCL3-5KO-F/PpDCL3-5KO-R, 5′- CCAAGCTTACTTCGACGGAATTCGACAGGGTT-3′/5′- AACTCGAGTAGTGATCACACGGTCACCAACCA-3′; PpDCL3-3KO-F/PpDCL3-3KO-F, 5′-CCAGATCTTACTCTTGGGTTTGGTTCTGGGCA-3′/5′-CCACGCGTATACCTTGCAGGCCCTCACCTAAT-3′) were utilized for amplification of 5′ upstream (970 bp) and 3′ downstream (1035 bp) fragments of *PpDCL3* ORF using the *P. patens* genomic DNA as a template; the resulting fragments were separately cloned into the pCR 4-TOPO TA cloning vector (Invitrogen, Carlsbad, CA, USA). The fragments were released by *Hind*III/*Xho*I and *Bgl*II/*Mlu*I restriction enzymes, respectively, and then ligated into the pUQ vector containing a hygromycin resistance cassette (generous gift from P-F Perroud in Washington University in St. Louis, USA) which was originally constructed on the basis of pUC19. The resulting disruption construct was digested with *Hind*III and *Mlu*I restriction enzymes to be linearized and precipitated. Polyethylene glycol-mediated protoplast transformation was performed as previously described [52] using a seven day-old protonemal tissue grown on cellophane-overlaid BCD media [30] supplemented with 50 mM ammonium tartrate under 16 hr days, 8 hr nights at 22°C. Targeting events were analyzed by PCR using genomic DNA, in which AF/AR (5′-GCGTTTGAATTTGGTTCCACCACC-3′/5′-AGATAGCTGGGCAATGGAATCCGA-3′) and BF/BR primer sets (5′-GGGTTTCGCTCATGTGTTGAGCAT-3′/5′-GCGAGCATTGTGCAAGTTTCCGTA-3′) were used for the analysis of 5′ and 3′ integration events, respectively. RT-PCR analyses were performed to identify the removal of *Ppdcl3* transcript with two sets of primers (CF/CR, 5′-TTGGTTTGTGGTGTGCATCCAAGG-3′/5′- GCAGTCATGGTGCATTGCTGTTCT-3′; DF/DR, 5′-GCTGCGAAGCGGGTTAATTGTCAT-3′/5′-CGTTGTTTACTGATGCCGTTCGCA-3′) and the accumulation of *hpt* transcript with a primer set (hptII F/hptII R, 5′-TGTTTATCGGCACTTTGCATCGGC-3′/5′- AGCTGCATCATCGAAATTGCCGTC-3′). RNA was isolated from 7 d-old protonemal tissue with Trizol reagent (Invitrogen, Carlsbad, CA, USA) and reverse transcribed using a SuperscriptIII reverse transcriptase (Invitrogen, Carlsbad, CA, USA) following the manufacturer's instructions. Thermocycling conditions were as follows: initial denaturation at 95°C for 5 min, followed by 35 cycles of 30 sec at 95°C, 1 min at 55°C, 30 sec at 72°C, and terminated by a 10 min-final extension at 72°C. *Ppubiquitin* primers (5′-ACTACCCTGAAGTTGTATAGTTCGG-3′/5′-CAAGTCACATTACTTCGCTGTCTAG-3′ were used as a control.

### Small RNA Sequencing and Data Analysis

Total RNA was extracted using Tri-Reagent (Sigma, St. Louis, MO, USA) per the manufacturer's instructions from 10-day old protonemal cultures of the wild-type, *Ppdcl3*-5, *Ppdcl3*-10, and *Pprdr6*-19 (kind gift of Tzahi Arazi). Cultures were grown as described above. Small RNA-enriched fractions were prepared by precipitating high molecular weight RNAs in the presence of 0.5 M NaCl and 10% (m/v) Polyethylene glycol (MW = ∼8,000) and recovering the supernatant. Pre-adenylated 3′ adapters (IDT, Coralville, IA) were added using T4 RNA ligase without exogenous ATP; the wild-type library used linker 1 (5′-AppCTGTAGGCACCATCAATddC-3′), both *Ppdcl3* libraries used linker 2 (5′-AppCACTCGGGCACCAAGGAddC-3′) and the *Pprdr6*-19 library used linker 3 (5′-AppTTTAACCGCGAATTCCAGddC-3′). Ligated products were gel purified and then ligated to a 5′ adapter composed of RNA (5′-GUUCAGAGUUCUACAGUCCGACGAUC-3′) using T4 RNA ligase with ATP. After gel purification, samples were reverse transcribed using oligos appropriate to the specific 3′ linker (1: 5′-ATTGATfGGTGCCTACAG-3′, 2: 5′-TCCTTGGTGCCCGAGTG-3′, 3: 5′-CTGGAATTCGCGGTTAAA-3′). cDNA libraries were then amplified using a constant 5′ oligo (5′-AATGATACGGCGACCACCGACAGGTTCAGAGTTCTACAGTCCGA-3′) and 3′ oligos specific for each of the linkers (1: 5′-CAAGCAGAAGACGGCATACGAATTGATGGTGCCTACAG-3′, 2: 5′-CAAGCAGAAGACGGCATACGATCCTTGGTGCCCGAGTG-3′, 3: 5′-CAAGCAGAAGACGGCATACGACTGGAATTCGCGGTTAAA-3′). Amplified libraries were then gel-purified and two samples were mixed containing equal amounts of the wild-type, a *Ppdcl3*, and the *Pprdr6*-19 library. Mixed samples were then sequenced using an Illumina genome analyzer by Fasteris, Inc. (Geneva, Switzerland). A detailed protocol is available upon request.

Raw FASTQ files were first processed to identify 3′ linker sequences as follows: The 5 nt sequence representing the first five bases of one of the 3′ linkers was searched for within an 8 nt window of the read (positions 19 through 26). If found, all linker bases were removed and the resulting small RNA read was assigned to the appropriate sample based upon the identity of the linker. If no 5 nt matches to any of the linkers was found, or if more than one was found, the read was discarded. Reads which exactly matched the sense strand of one or more rRNAs (nuclear, plastid, or mitochondrial) were then discarded. The remaining reads were mapped to the *P. patens* version 1.1 draft genome assembly using oligomap [53], accepting only perfect alignments; reads which failed to match perfectly to at least one position in the genome were also discarded to leave the final datasets used for analyses. Normalized reads per million (NRPM) was calculated as follows: First, the repeat normalized abundance for each small RNA was calculated by dividing the raw number of reads by the number of perfect matches to the genome. This value was then scaled by dividing by the total number of genome-matched non-rRNA reads in the sample, and multiplying the result by one million. Data have been deposited at NCBI GEO (GSE12468).

### Analyses of 5-Methyl Cytosine

Genomic DNA was isolated from seven day-old *P. patens* protonemal tissues using a Nucleon Phytopure Plant DNA extraction kit (Amersham Biosciences, Piscataway, NJ, USA) following the manufacturer's instructions. Bisulfite conversion of 500 ng of genomic DNA was performed using an EZ DNA Methylation kit (Zymo Research, Orange, CA, USA) following the manufacturer's protocol. Regions analyzed were amplified from 2 µl of bisulfite converted DNA using sets of specific primers (Table S4). The primers were designed to have A or T at the 3′ end, a 30–50% GC content, a 200–400 bp amplicon size, a maximum of three nucleotides degeneracy, and unique mapping on the genome. PCR products were cloned into a pCR 4-TOPO vector (Invitrogen, Carlsbad, CA, USA) following the manufacturer's instructions, and 8–28 independent clones were sequenced and analyzed. Details of bisulfite sequencing results can be found in Table S5. For the *Mcr*BC assay, genomic DNA (500 ng) was treated with 20 units of *McrBC* (New England Biolabs Inc., Ipswich, MA, USA) for 4 h at 37°C. Amplification was performed using sets of specific primers (Table S4).

### RT-PCR

Total RNAs were extracted from 10 d-old protonemal tissues using an RNeasy Plant Mini kit (Qiagen Inc., Valencia, CA, USA), followed by a DNase treatment (Qiagen Inc., Valencia, CA, USA) following the manufacturer's protocol except that the length of incubation was 20 minutes. 500 ng of the RNAs were converted to cDNA using Superscript III reverse transcriptase (Invitrogen, Carlsbad, CA, USA) primed with random hexamers (New England Biolabs Inc., Ipswich, MA, USA) following the manufacturer's instruction. Thermocycling conditions were as follows: initial denaturation at 95°C for 5 min, followed by 35 (for *PpRT3* and *PpRT6*) or 50 cycles (for all other small RNA loci) of 30 sec at 95°C, 1 min at 50°C, 30 sec at 72°C, and terminated by a 10 min-final extension at 72°C. The same primers as in the *Mcr*BC assay were used for amplification of small RNA loci, whereas sets of specific primers were used for *PpRT3* and *PpRT6* (Table S4).

### RNA Blot Analysis

Total RNA was separated in a 12% denaturing polyacrylamide gel containing 8.3 M urea in TBE buffer, and electroblotted onto nylon membranes for 1 h at 400 mA. Radiolabeled probes were generated by end-labeling of DNA oligonucleotides complementary to miRNA, tasiRNA sequences and the U6 snRNA control with γ^32^P-ATP using T4 polynucleotide kinase. Blot hybridization was carried out in 0.05 M sodium phosphate (pH 7.2), 1 mM EDTA, 6×SSC, 1×Denhardt's, 5% SDS. Blots were washed 2–3 times with 2×SSC, 0.2% SDS and one time with 1×SSC, 0.1% SDS. Blots were hybridized and washed at temperatures 10°C below the T_m_ of the oligonucleotide. The sequences of the oligonucleotides used for the detection of small RNAs are listed in Table S4.

## Supporting Information

Figure S1 Targeted deletion of *P. patens PpDCL3*. A) Schematic of homologous recombination scheme. Labeled arrows indicate oligos used for PCR and RT-PCR analyses. Solid rectangles indicate exons, lines indicate introns. CaMV: Caulifolwer Mosaic Virus, *hptII*: hygromycin phosphotransferase II gene. Not to scale. (B) PCR of genomic DNA using the indicated primer pairs. (C) RT-PCR analyses of gene expression using the indicated primer pairs.
(0.59 MB PDF)

Figure S2 Distribution of small RNAs from *Pp23SR* loci by length and 5′ nucleotide. Each graph represents the *Pp23SR* matched portion of the indicated small RNA library. A, U, G, and C refer to the identity of the 5′ nucleotide. NRPM: Normalized reads per million.
(0.19 MB PDF)

Figure S3 Distribution of all genome-mapped small RNAs by length and 5′ nucleotide. (A) Each graph represents the indicated small RNA library, counted by abundance (number of reads). A, U, G, and C refer to the identity of the 5′ nucleotide. (B) As in A, except tallied by uniquely obtained sequences regardless of abundance.
(0.26 MB PDF)

Figure S4 RT-PCR on the selected small RNA producing loci. *PpActin* primer was used a control.
(1.23 MB PDF)

Table S1 Novel miRNAs from *Physcomitrella patens*.
(0.06 MB XLS)

Table S2 *Pp21SR* loci.
(0.04 MB XLS)

Table S3 *Pp23SR* loci.
(0.05 MB XLS)

Table S4 Primer sets used for amplification of small RNA loci.
(0.07 MB DOC)

Table S5 Bisulfite sequencing data.
(0.11 MB DOC)
