# Supplementary material for: Physcomitrella patens DCL3 Is Required for 22–24 nt siRNA Accumulation, Suppression of Retrotransposon-Derived Transcripts, and Normal Development
Source: PLoS Genet. 2008 Dec 19;4(12):e1000314. doi: 10.1371/journal.pgen.1000314 (PMC2600652; doi:10.1371/journal.pgen.1000314)
Supplement: Figure S1 — Targeted deletion of P. patens PpDCL3. A) Schematic of homologous recombination scheme. Labeled arrows indicate oligos used for PCR and RT-PCR analyses. Solid rectangles indicate exons, lines indicate introns. CaMV: Caulifolwer Mosaic Virus, hptII: hygromycin phosphotransferase II gene. Not to scale. (B) PCR of genomic DNA using the indicated primer pairs. (C) RT-PCR analyses of gene expression using the indicated primer pairs. (0.59 MB PDF) [file pgen.1000314.s001.pdf]

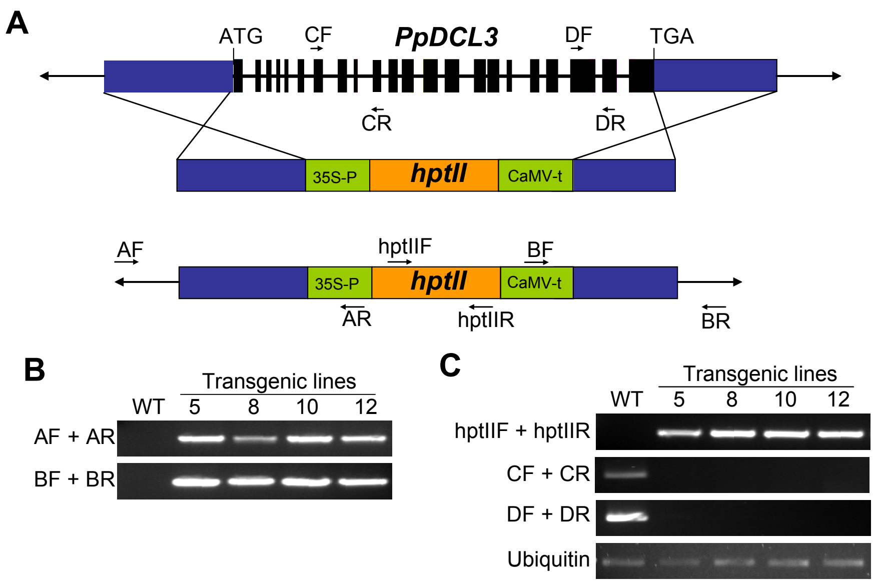

Figure S1. Targeted deletion of *P. patens PpDCL3*. (A) Schematic of homologous recombination scheme. Labeled arrows indicate oligos used for PCR and RT-PCR analyses. Solid rectangles indicate exons, lines indicate introns. CaMV: Cauliflower Mosaic Virus, *hptII*: hygromycin phosphotransferase II gene. Not to scale. (B) PCR of genomic DNA using the indicated primer pairs. (C) RT-PCR analyses of gene expression using the indicated primer pairs.
