# Supplementary material for: Physcomitrella patens DCL3 Is Required for 22–24 nt siRNA Accumulation, Suppression of Retrotransposon-Derived Transcripts, and Normal Development
Source: PLoS Genet. 2008 Dec 19;4(12):e1000314. doi: 10.1371/journal.pgen.1000314 (PMC2600652; doi:10.1371/journal.pgen.1000314)
Supplement: Figure S2 — Distribution of small RNAs from Pp23SR loci by length and 5′ nucleotide. Each graph represents the Pp23SR matched portion of the indicated small RNA library. A, U, G, and C refer to the identity of the 5′ nucleotide. NRPM: Normalized reads per million. (0.19 MB PDF) [file pgen.1000314.s002.pdf]

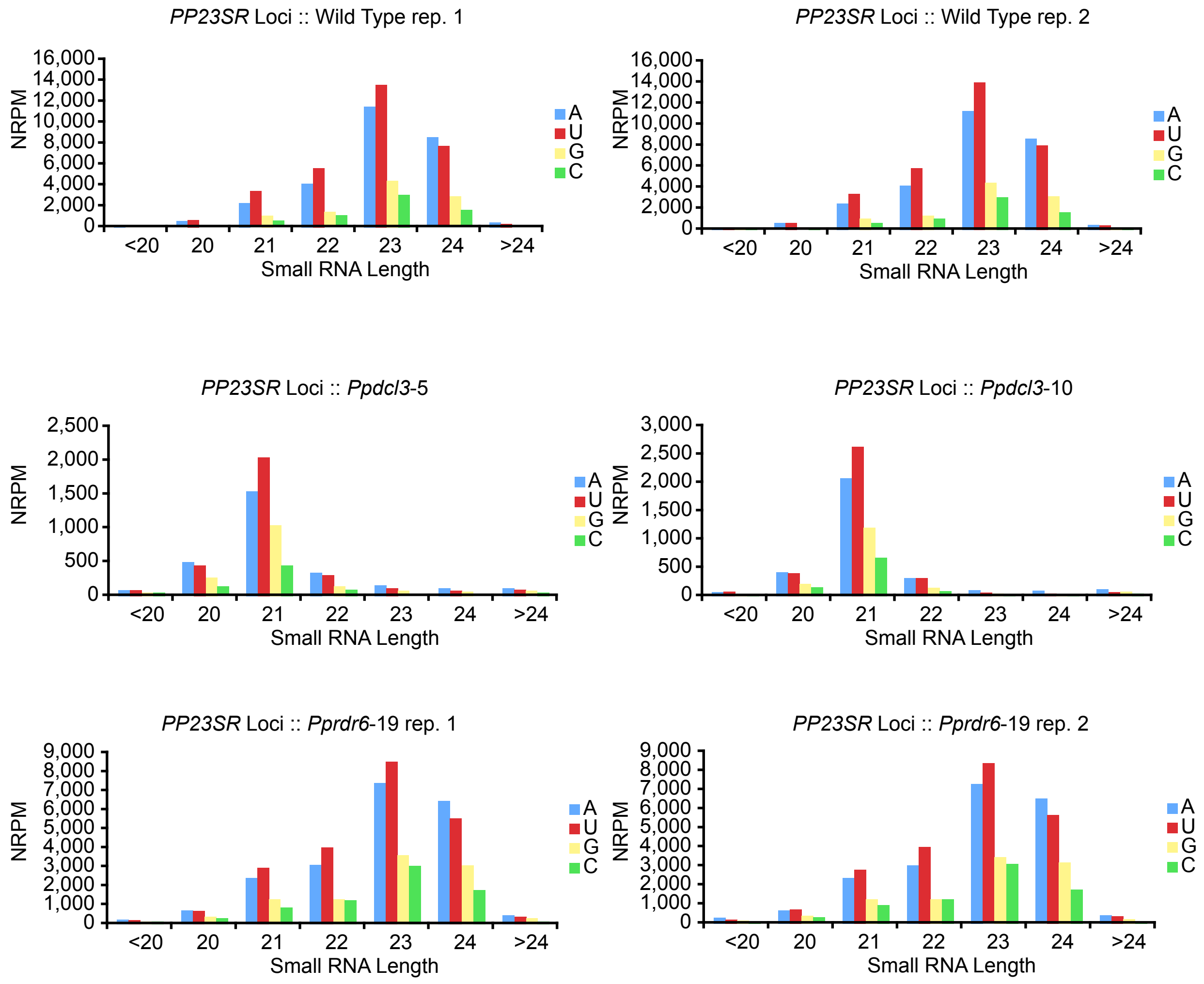

Figure S2. Distribution of small RNAs from *Pp23SR* loci by length and 5' nucleotide. Each graph represents the *Pp23SR* matched portion of the indicated small RNA library. A, U, G, and C refer to the identity of the 5' nucleotide. NRPM: Normalized reads per million.
