# Supplementary material for: Physcomitrella patens DCL3 Is Required for 22–24 nt siRNA Accumulation, Suppression of Retrotransposon-Derived Transcripts, and Normal Development
Source: PLoS Genet. 2008 Dec 19;4(12):e1000314. doi: 10.1371/journal.pgen.1000314 (PMC2600652; doi:10.1371/journal.pgen.1000314)
Supplement: Figure S3 — Distribution of all genome-mapped small RNAs by length and 5′ nucleotide. (A) Each graph represents the indicated small RNA library, counted by abundance (number of reads). A, U, G, and C refer to the identity of the 5′ nucleotide. (B) As in A, except tallied by uniquely obtained sequences regardless of abundance. (0.26 MB PDF) [file pgen.1000314.s003.pdf]

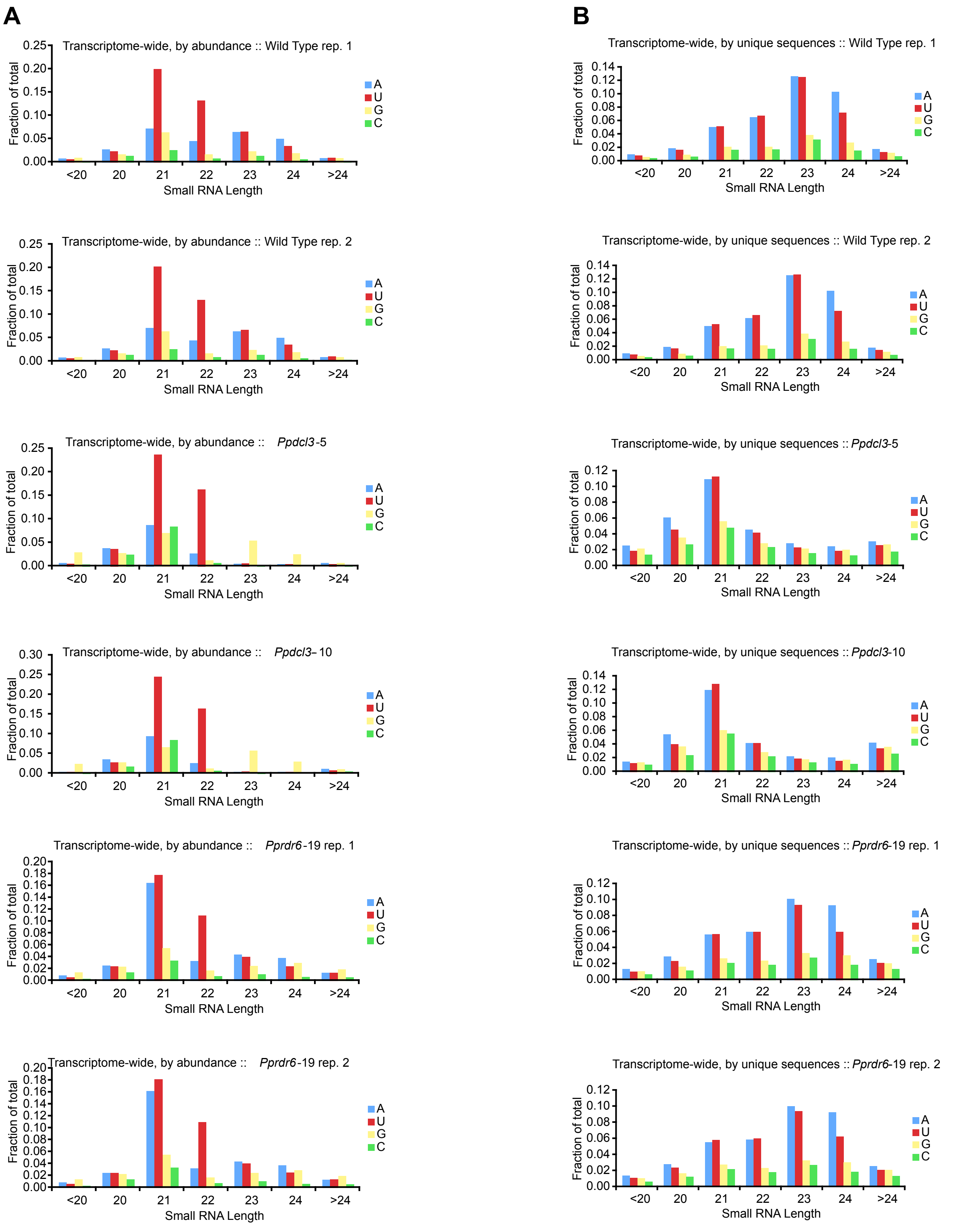

Figure S3. Distribution of all genome-mapped small RNAs by length and 5' nucleotide. (A) Each graph represents the indicated small RNA library, counted by abundance (number of reads). A, U, G, and C refer to the identity of the 5' nucleotide. (B) As in A, except tallied by uniquely obtained sequences regardless of abundance.
