# Supplementary figures and images for: Physcomitrella patens DCL3 Is Required for 22–24 nt siRNA Accumulation, Suppression of Retrotransposon-Derived Transcripts, and Normal Development
Source: PLoS Genet. 2008 Dec 19;4(12):e1000314. doi: 10.1371/journal.pgen.1000314 (PMC2600652; doi:10.1371/journal.pgen.1000314)

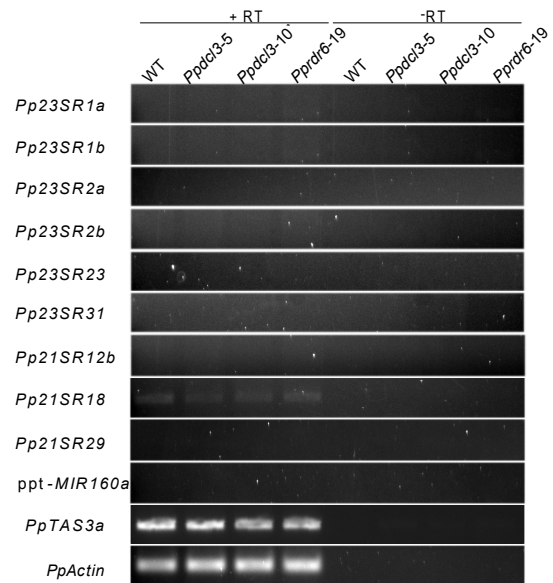

**Figure S4. RT-PCR on the selected small RNA producing loci.**  
*PpActin* primer was used a control.

Supplement: Figure S4 — RT-PCR on the selected small RNA producing loci. PpActin primer was used a control. (1.23 MB PDF) [file pgen.1000314.s004.pdf]
