# Supplementary material for: Physcomitrella patens DCL3 Is Required for 22–24 nt siRNA Accumulation, Suppression of Retrotransposon-Derived Transcripts, and Normal Development
Source: PLoS Genet. 2008 Dec 19;4(12):e1000314. doi: 10.1371/journal.pgen.1000314 (PMC2600652; doi:10.1371/journal.pgen.1000314)
Supplement: Table S4 — Primer sets used for amplification of small RNA loci. (0.07 MB DOC) [file pgen.1000314.s008.doc]

**Table S4**. Primer sets used

| **Exp^1^** | **Locus** | **Region** | **Forward Sequences (5'→3') / Reverse Sequences (5'→3')** | **Position** |
| --- | --- | --- | --- | --- |
| Bisulfite  sequencing | *Pp23SR1* | a | TTTTTCCAATATCCTCTCCCTTCT / TYGATGATGTYTTTGGAAGYATGA | Scaffold 83:387910-388288 |
|  |  | b | AATCCAATCCTACACCCAAAATTA / ATTGYGYYATAGAYAAAAGTGGTA | Scaffold 83:390297-390628 |
|  | *Pp23SR2* | a | TGAGATAGYATYTTTGGAGGGTGA / CATRCATCTRTARCCTTTTTRCAA | Scaffold 287: 356430-356708 |
|  |  | b | TRCCCATRTCATTTTTTRCAAAAA / GGAGGATAAYGTTGAAAAYGGTTA | Scaffold 287: 356670-357098 |
|  | *Pp23SR23* |  | TTAGAYGAYYATGGAGAGGATYAT / ARTCCTTTTCTCAACARCTTCAAA | Scaffold 179: 234677-234985 |
|  | *Pp23SR31* |  | TTCAATATCCTCCCCCTCCTATCT / AGGYTAAGGAAYAATATGATGGYA | Scaffold 117: 1192783-1193180 |
|  | *Pp23SR35* |  | CAATRAACATRTTAATCACCTCAT / GGAYGATTTGTTTGTAAAYYATYA | Scaffold 65: 446953-447246 |
|  | *Pp21SR12* | a | AAGGGGATTGTYAAGYAGAAAGYA / AACTTCATTTCATACAAACCCCCA | Scaffold 17: 49376-49681 |
|  |  | b | TCTTTGTGGGATGTTGATAAAAGA / CCACCTACCTCATAAAATATTACA | Scaffold 17: 49704-50051 |
|  | *Pp21SR18* |  | TCCTATACAAATACCAACRTCTTA / AATTGGGGTGYYTAATGGATTGGA | Scaffold 132: 926578-926899 |
|  | *Pp21SR29* |  | ACCACTTTTCCCTCTTCTTTTACA / GGGTTAAGTATYGGTAATAYTTGA | Scaffold 446: 176396-176722 |
|  | ppt-*MIR160a* |  | RCTATRCARCTCRACAATCACTTT / GGAGATGATYTYGATGTTTGATTA | Scaffold 104: 1047254-1047338 |
|  | *PpTAS3a* |  | GTTAGYGGGGTGTTAAGYATTTGA / CAACAAAACTACCTCTARTCTTRA | Scaffold 61: 113267-113506 |
| *Mcr*BC assay | *Pp23SR1* | a | TTTTTCCAATATCCTCTCCCTTCT / TCGATGATGTCTTTGGAAGCATGA | Scaffold 83:387910-388288 |
|  |  | b | AATCCAATCCTACACCCAAAATTA / ATTGCGCCATAGACAAAAGTGGTA | Scaffold 83:390297-390628 |
|  | *Pp23SR2* | a | TGAGATAGCATCTTTGGAGGGTGA / CATGCATCTGTAGCCTTTTTGCAA | Scaffold 287: 356430-356708 |
|  |  | b | TGCCCATGTCATTTTTTGCAAAAA / GGAGGATAACGTTGAAAACGGTTA | Scaffold 287: 356670-357098 |
|  | *Pp23SR23* |  | TTAGACGACCATGGAGAGGATCAT / AGTCCTTTTCTCAACAGCTTCAAA | Scaffold 179:234677-234985 |
|  | *Pp23SR31* |  | TTCAATATCCTCCCCCTCCTATCT / AGGCTAAGGAACAATATGATGGCA | Scaffold 179: 234677-234985 |
|  | *Pp23SR35* |  | CAATGAACATGTTAATCACCTCAT / GGACGATTTGTTTGTAAACCATCA | Scaffold 65: 446953-447246 |
|  | *Pp21SR12* | a | AAGGGGATTGTCAAGCAGAAAGCA / AACTTCATTTCATACAAACCCCCA | Scaffold 17: 49376-49681 |
|  |  | b | TCTTTGTGGGATGTTGATAAAAGA / CCACCTACCTCATAAAATATTACA | Scaffold 17: 49704-50051 |
|  | *Pp21SR18* |  | TCCTATACAAATACCAACGTCTTA / AATTGGGGTGCCTAATGGATTGGA | Scaffold 132: 926578-926899 |
|  | *Pp21SR29* |  | ACCACTTTTCCCTCTTCTTTTACA / GGGTTAAGTATCGGTAATACTTGA | Scaffold 446: 176396-176722 |
|  | ppt-*MIR160a* |  | GCTATGCAGCTCGACAATCACTTT / GGAGATGATCTCGATGTTTGATTA | Scaffold 104: 1047254-1047338 |
|  | *PpTAS3a* |  | GTTAGCGGGGTGTTAAGCATTTGA / CAACAAAACTACCTCTAGTCTTGA | Scaffold 61: 113267-113506 |
| RT-PCR | *RT3* |  | AACCATGGTCTTCTRTTTCTATGGAYTTCATCA / CCAAAATCTTGATACAAATTGAGT |  |
|  | *RT6* |  | ACATCGAYGAGAATCTAGCCAAGAAYTTYA / AAAATATCATCYARRTAGATGACAACAAA |  |
|  | *actin* |  | ATCTGGAATGGTCAAGGCCGGTTT / TCATCTTCTCCCTGTTCGCCTTCG |  |
| Small RNA blot probes | miR390c |  | GGCGCTATCCCTCCTGAGCTC | scaffold_264: 204264-204398 |
|  | *PpTAS3a*-3p+3-5+_1 |  | TGAAGCACTCATCACACCCTA | scaffold_61: 113349-113329 |
|  | *PpTAS3a*-3p+1-3-_1 |  | TGCCCACCTACCCTTGTGATA | scaffold_61: 113371-113391 |
|  | U6 snRNA |  | GGGGCCATGCTAATCTTCTCTG |  |

**^1^**Experimental applications
