# Supplementary material for: Physcomitrella patens DCL3 Is Required for 22–24 nt siRNA Accumulation, Suppression of Retrotransposon-Derived Transcripts, and Normal Development
Source: PLoS Genet. 2008 Dec 19;4(12):e1000314. doi: 10.1371/journal.pgen.1000314 (PMC2600652; doi:10.1371/journal.pgen.1000314)
Supplement: Table S5 — Bisulfite sequencing data. (0.11 MB DOC) [file pgen.1000314.s009.doc]

**Table S5. Bisulfite Sequencing Data**

**Wild Type**

|  | **CG** | | | **CHG** | | | **CHH** | | | Clone #^4^ |
| --- | --- | --- | --- | --- | --- | --- | --- | --- | --- | --- |
|  | (-)^1^ | (+)^2^ | Ratio^3^ | (-) | (+) | Ratio | (-) | (+) | Ratio |  |
| ***Pp23SR1-a*** | 222 | 155 | **69.8** | 217 | 192 | **88.5** | 1351 | 737 | **54.6** | 28 |
| ***Pp23SR1-b*** | 255 | 241 | **94.5** | 34 | 31 | **91.2** | 816 | 580 | **71.1** | 17 |
| ***Pp23SR2-a*** | 24 | 9 | **37.5** | 36 | 15 | **41.7** | 444 | 114 | **25.7** | 12 |
| ***Pp23SR2-b*** | 66 | 45 | **68.2** | 44 | 44 | **100.0** | 736 | 653 | **88.7** | 11 |
| ***Pp23SR23*** | 47 | 30 | **63.8** | 32 | 19 | **59.4** | 582 | 305 | **52.4** | 16 |
| ***Pp23SR31*** | 64 | 54 | **84.4** | 80 | 70 | **87.5** | 438 | 243 | **55.5** | 8 |
| ***Pp23SR35*** | 133 | 87 | **65.4** | 76 | 34 | **44.7** | 874 | 255 | **29.2** | 19 |
| ***Pp21SR12-a*** | 96 | 0 | **0.0** | 180 | 1 | **0.6** | 480 | 2 | **0.4** | 12 |
| ***Pp21SR12-b*** | 143 | 26 | **18.2** | 104 | 23 | **22.1** | 689 | 165 | **23.9** | 13 |
| ***Pp21SR18*** | 24 | 22 | **91.7** | 24 | 24 | **100.0** | 324 | 156 | **48.1** | 12 |
| ***Pp21SR29*** | 133 | 96 | **72.18** | 114 | 79 | **69.30** | 551 | 374 | **67.88** | 19 |
| **ppt-*MIR160a*** | 120 | 21 | **17.5** | 105 | 13 | **12.4** | 465 | 51 | **11.0** | 15 |
| ***PpTAS3a*** | 90 | 7 | **7.8** | 54 | 0 | **0.0** | 378 | 22 | **5.8** | 9 |

***Ppdcl3-5***

|  | **CG** | | | **CHG** | | | **CHH** | | | Clone #^4^ |
| --- | --- | --- | --- | --- | --- | --- | --- | --- | --- | --- |
|  | (-)^1^ | (+)^2^ | Ratio^3^ | (-) | (+) | Ratio | (-) | (+) | Ratio |  |
| ***Pp23SR1-a*** | 75 | 56 | **74.7** | 72 | 68 | **94.4** | 450 | 156 | **34.7** | 9 |
| ***Pp23SR1-b*** | 142 | 128 | **90.1** | 19 | 11 | **57.9** | 453 | 207 | **45.7** | 10 |
| ***Pp23SR2-a*** | 16 | 6 | **37.5** | 22 | 10 | **45.5** | 287 | 138 | **48.1** | 8 |
| ***Pp23SR2-b*** | 64 | 43 | **67.2** | 42 | 41 | **97.6** | 712 | 573 | **80.5** | 11 |
| ***Pp23SR23*** | 30 | 22 | **73.3** | 20 | 14 | **70.0** | 370 | 217 | **58.6** | 10 |
| ***Pp23SR31*** | 86 | 78 | **90.70** | 105 | 94 | **89.52** | 586 | 254 | **43.34** | 11 |
| ***Pp23SR35*** | 55 | 38 | **69.1** | 32 | 17 | **53.1** | 354 | 145 | **41.0** | 8 |
| ***Pp21SR12-a*** | 72 | 2 | **2.8** | 134 | 2 | **1.5** | 361 | 3 | **0.8** | 9 |
| ***Pp21SR12-b*** | 121 | 30 | **24.8** | 86 | 25 | **29.1** | 581 | 206 | **35.5** | 11 |
| ***Pp21SR18*** | 20 | 17 | **85.0** | 16 | 16 | **100.0** | 274 | 96 | **35.0** | 10 |
| ***Pp21SR29*** | 119 | 104 | **87.39** | 102 | 75 | **73.53** | 493 | 311 | **63.08** | 17 |
| **ppt-*MIR160a*** | 70 | 11 | **15.7** | 56 | 5 | **8.9** | 268 | 17 | **6.3** | 9 |
| ***PpTAS3a*** | 90 | 7 | **7.8** | 54 | 0 | **0.0** | 378 | 21 | **5.6** | 9 |

***Ppdcl3-10***

|  | **CG** | | | **CHG** | | | **CHH** | | | Clone #^4^ |
| --- | --- | --- | --- | --- | --- | --- | --- | --- | --- | --- |
|  | (-)^1^ | (+)^2^ | Ratio^3^ | (-) | (+) | Ratio | (-) | (+) | Ratio |  |
| ***Pp23SR1-a*** | 74 | 57 | **77.0** | 72 | 66 | **91.7** | 450 | 153 | **34.0** | 9 |
| ***Pp23SR1-b*** | 149 | 135 | **90.6** | 20 | 13 | **65.0** | 478 | 144 | **30.1** | 10 |
| ***Pp23SR2-a*** | 16 | 7 | **43.8** | 24 | 13 | **54.2** | 296 | 112 | **37.8** | 8 |
| ***Pp23SR2-b*** | 78 | 54 | **69.2** | 52 | 51 | **98.1** | 868 | 549 | **63.2** | 13 |
| ***Pp23SR23*** | 32 | 21 | **65.6** | 22 | 19 | **86.4** | 407 | 247 | **60.7** | 11 |
| ***Pp23SR31*** | 80 | 68 | **85.0** | 100 | 83 | **83.0** | 547 | 242 | **44.2** | 10 |
| ***Pp23SR35*** | 77 | 55 | **71.4** | 44 | 24 | **54.5** | 497 | 258 | **51.9** | 11 |
| ***Pp21SR12-a*** | 72 | 0 | **0.0** | 135 | 0 | **0.0** | 360 | 2 | **0.6** | 9 |
| ***Pp21SR12-b*** | 110 | 30 | **27.3** | 80 | 29 | **36.3** | 526 | 183 | **34.8** | 10 |
| ***Pp21SR18*** | 22 | 19 | **86.4** | 22 | 22 | **100.0** | 297 | 98 | **33.0** | 11 |
| ***Pp21SR29*** | 70 | 47 | **67.1** | 60 | 32 | **53.3** | 290 | 147 | **50.7** | 10 |
| **ppt-*MIR160a*** | 78 | 13 | **16.7** | 65 | 7 | **10.8** | 298 | 15 | **5.0** | 10 |
| ***PpTAS3a*** | 100 | 9 | **9.0** | 60 | 0 | **0.0** | 420 | 17 | **4.0** | 10 |

^1^ Number of C’s according to draft genome sequence

^2^ Number of C’s observed from bisulfite treated samples (representing 5mC)

^3^ Presented as a percentage

^4^ Number of clones sequenced
